# Finite element model predicts micromotion-induced strain profiles that correlate with the functional performance of Utah arrays in humans and non-human primates

**DOI:** 10.1088/1741-2552/ae1bda

**Published:** 2025-11-18

**Authors:** Adam M Forrest, Nicolas G Kunigk, Jennifer L Collinger, Robert A Gaunt, Xing Chen, Jonathan P Vande Geest, Takashi D Y Kozai

**Affiliations:** 1 Department of Bioengineering, University of Pittsburgh, Pittsburgh, PA, United States of America; 2 Center for Neural Basis of Cognition, Pittsburgh, PA, United States of America; 3 Rehab Neural Engineering Labs, University of Pittsburgh, Pittsburgh, PA, United States of America; 4 Department of Physical Medicine and Rehabilitation, University of Pittsburgh, Pittsburgh, PA, United States of America; 5 Biomedical Engineering Department, Carnegie Mellon University, Pittsburgh, PA, United States of America; 6 Department of Vision & Cognition, Netherlands Institute for Neuroscience, Amsterdam, The Netherlands; 7 Department of Ophthalmology, University of Pittsburgh, Pittsburgh, PA, United States of America; 8 McGowan Institute for Regenerative Medicine, University of Pittsburgh, Pittsburgh, PA, United States of America; 9 Department of Mechanical Engineering and Material Science, University of Pittsburgh, Pittsburgh, PA, United States of America; 10 Center for Neuroscience, University of Pittsburgh, Pittsburgh, PA, United States of America; 11 Neuroscience Institute, Carnegie Mellon University, Pittsburgh, PA, United States of America

**Keywords:** brain–computer interface, implant mechanics, microelectrode array, chronic signal performance, impedance, signal-to-noise ratio, mechanosensation

## Abstract

*Objective.* Utah arrays are widely used in both humans and non-human primates (NHPs) for intracortical brain–computer interfaces, primarily for detecting electrical signals from cortical tissue to decode motor commands. Recently, these arrays have also been applied to deliver electrical stimulation aimed at restoring sensory functions. A key challenge limiting their longevity is the micromotion between the array and cortical tissue, which may induce mechanical strain in surrounding tissue and contribute to performance decline. This strain, due to mechanical mismatch, can exacerbate glial scarring around the implant, reducing the efficacy of Utah arrays in recording neuronal activity and delivering electrical stimulation. *Approach.* To investigate this, we employed a finite element model to predict tissue strains resulting from micromotion. *Main results.* Our findings indicated that strain profiles around edge and corner electrodes were greater than those around interior shanks, affecting both maximum and average strains within 50 *µ*m of the electrode tip. We then correlated these predicted tissue strains with *in-vivo* electrode performance metrics. We found negative correlations between 1 kHz impedance and tissue strains in human motor arrays and NHP area V4 arrays at 1 month, 1 year, and 2 years post-implantation. In human motor arrays, the peak-to-peak waveform voltage and signal-to-noise ratio (SNR) of spontaneous activity were also negatively correlated with strain. Conversely, we observed a positive correlation between the evoked SNR of multi-unit activity and strain in NHP area V4 arrays. *Significance.* This study establishes a spatial dependence of electrode performance in Utah arrays that correlates with tissue strain.

## 1. Introduction

Brain–computer interfaces (BCIs) are advanced technologies that can be integrated into therapeutic strategies to restore motor and sensory function in individuals with central nervous system injuries and disorders [1, 2]. BCIs have previously employed arrays of ∼100 intracortical microelectrode shanks implanted in the motor cortex to capture neural signals by recording action potentials from surrounding neurons, which can then be decoded to control devices, such as robotic limbs [3–7]. Additionally, in the somatosensory and visual cortices, microelectrode arrays have been used to deliver electrical stimulation and restore artificial tactile and visual sensations [8–13]. A critical consideration for implanted BCIs is the longevity of these devices [14–21]. While intracortical arrays can record action potentials for years, their ability to record from nearby neurons is highly variable [22, 23] and diminishes over time [15, 19, 24–27]. Although researchers have investigated new electrode shank designs to improve longevity [28–38], the influence of neighboring shanks on each other remains underexplored.

Designs of microelectrode arrays influence the degree of tissue response following microelectrode implantation, in turn impacting device longevity. Acute tissue damage during implantation disrupts the blood-brain barrier, reducing nutrient delivery to the disrupted tissue, releasing plasma proteins into the parenchyma, and triggering an inflammatory response that leads to an electrically insulating glial scar around the microelectrodes [39–43]. Activated microglia and astrocytes at the glial scar release cytokines that promote a neurodegenerative environment at the electrode–tissue interface further reducing neuronal activity and signal detectability [44–52]. This neuroinflammation is exacerbated by strain concentrations in the tissue caused by micromotions of the electrode relative to the brain [53–55]. Behavioral head movements, respiration, and blood vessel pulsations contribute to brain movement [56–60]. Because typical electrodes are 8 orders of magnitude stiffer than brain tissue, this relative motion generates mechanical strain in the surrounding parenchyma [61], which increases scarring around implants and impairs BCI performance [62–69].

Mechanical strain can activate neurons and glial cells via mechanosensitive ion channels [53, 54, 70]. In microglia, these channels can direct migration towards stiffer substrates and prompt phagocytic activity of cellular debris [58, 59, 71, 72]. Similarly, astrocytic inflammatory responses are mediated through mechanosensitive ion channels [43, 73, 74]. Reducing mechanical strain in the surrounding tissue could minimize glial activation and scarring, thereby promoting an improved electrode–tissue interface [66, 67, 75, 76]. Neurons can be directly damaged by mechanical injury during implantation, which is evidenced by altered cellular morphology and elevated calcium levels, potentially impacting the ability to reliably record neuronal activity [66, 77]. Damage to blood vessels may also reduce metabolic support to nearby neurons and contribute to local neuronal dysfunction, hampering signal acquisition and BCI performance [78–80]. As the number of shanks increases [37, 81–83], the cumulative mechanical strain within the tissue can become more pronounced [34, 84], exacerbating these effects by further compromising vascular integrity, increasing the extent of glial scarring, and reducing the overall functionality and longevity of microelectrodes. This heightened strain may also lead to greater variability in signal acquisition across the array, complicating the interpretation of neural signals and the effectiveness of the BCI system.

Finite element models (FEMs) have shown that linear electrodes, characterized by a single shank with multiple electrode sites along its length, exhibit high strain profiles near the electrode tip due to micromotions [85–91]. This strain concentration has been linked to neuroinflammation and the foreign-body response, limiting the longevity and reliability of these devices. However, strain profiles around bed-of-needle planar-style Utah arrays, characterized by rectangular or square base substrates with equidistantly spaced electrodes, have not been thoroughly characterized. Shank-to-shank distance alters tissue strain profiles during insertion and contributes to astrogliosis [34, 92], but the impact of micromotions on strain distributions in Utah arrays remains unclear. Understanding how micromotion-induced tissue strains affect device longevity is crucial for designing long-lasting multi-shank microelectrode arrays.

This study investigates how Utah array geometry influences tissue strain and recording performance. Using a FEM, micromotion-derived strain was predicted throughout the surrounding tissue and then correlated with device performance metrics, including impedance, peak-to-peak waveform voltage (PTPV), and signal-to-noise ratio (SNR) in both humans and non-human primates (NHPs). The distinct geometry of Utah arrays suggests that tissue strain profiles will vary based on electrode location within the array. For example, edge and corner electrodes may experience different mechanical interactions with surrounding brain tissue compared to those in the interior. The array’s geometry could also influence vascular and ischemic injuries, which are known to impact device performance. Vascular injury may be more pronounced at the edges, where blood vessels are more susceptible to implantation damage due to tissue dimpling [37, 93]. Conversely, ischemic injury could be greater at the array’s center, where the distance to undamaged blood supply is greater. While changes in performance metrics over the implant’s lifetime have been documented, the spatial dependence of these measures within Utah arrays remains underexplored. Addressing this gap will enhance understanding of how electrode positioning within the array impacts overall device performance, longevity, and the health of surrounding tissue.

## 2. Methods

### 2.1. FEM

#### 2.1.1. Geometry

**Figure 1.**
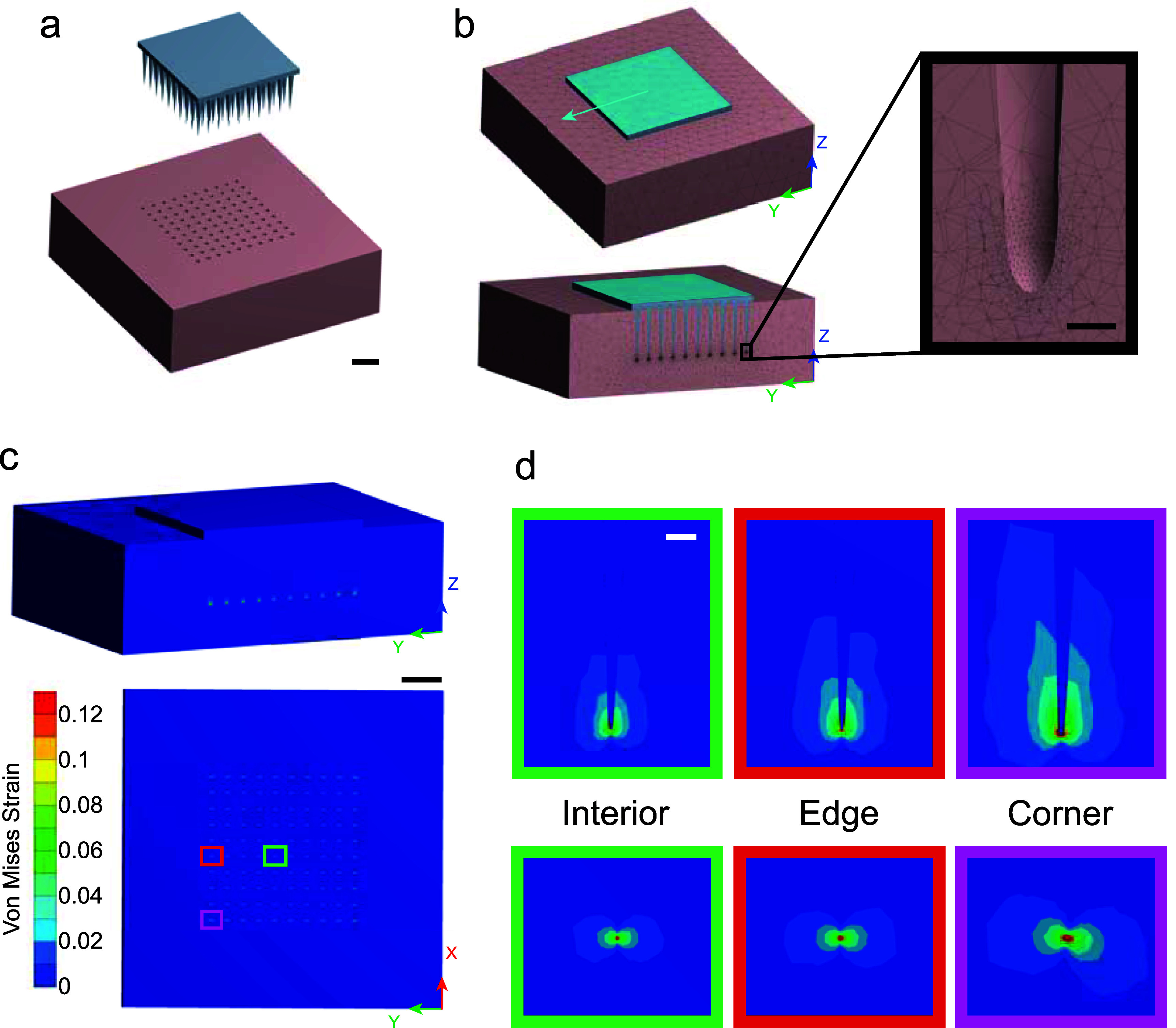
Finite element model predicts strains in brain tissue surrounding implanted Utah array that result from micromotions. (a) Geometry of 10 × 10 array (top) and brain tissue (bottom). Scale bar = 1 mm. (b) Mesh of 10 × 10 array embedded in cortical tissue with applied boundary conditions. The top face of the array was prescribed a displacement of 10 *µ*m in the *Y* direction. The bottom face of the brain tissue was held fixed. Sliced model (bottom) shows the fine mesh surrounding the tips of the shanks, further shown in inset (right). Scale bar = 10 *µ*m. (c) Contour plots of von Mises strain. Model was sliced in *YZ* plane (top) and *XY* plane (bottom) to uncover the tips of the electrodes. Scale bar = 1 mm. (d) Contour plots surrounding an example interior, edge, and corner electrode. Color bar is same as in (c). Scale bar = 100 *µ*m.

Electrode array geometries were designed to replicate the dimensions of the Utah arrays used in the electrophysiological recordings (Blackrock Neurotech). Three array geometries were modeled: 100-electode arrays in a 10 × 10 grid, 60-electrode arrays in a 10 × 6 grid, and 64 electrode arrays in an 8 × 8 grid. Each grid of electrodes had the same 400 *µ*m spacing. 10 × 10 arrays were modeled with a square 4 mm × 4 mm × 0.2 mm base substrate. Electrode shanks were defined by tapering from a 150 *µ*m × 150 *µ*m square at the base to a 10 *µ*m diameter circle at 1.49 mm from the base. To form the tip, a quarter ellipse with a major radius of 10 *µ*m and a minor radius of 5 *µ*m was revolved, resulting in a total shank length of 1.5 mm. 10 × 6 and 8 × 8 arrays were modeled in the same fashion, but utilized a rectangular 4 mm × 2.4 mm × 0.2 mm base substrate and a square 3.2 mm × 3.2 mm × 0.2 mm base substrate, respectively. Surrounding cortical brain tissue was modeled as a rectangular prism extending 1 mm beyond the electrode tips and 2 mm outward from the base on all sides. This tissue was modeled as a separate entity in bonded contact with the electrode shanks and substrate (figure 1(a)). The bonded contact is intended to simulate strong adhesion of cortical tissue to the implanted array.

Additional geometries were generated with the electrodes terminating to an exact point. These models were untenable as the FEMs would not reach mesh convergence due to infinite point stresses at the shank tip.

#### 2.1.2. Material properties

The electrode array was assumed to be silicon with linear isotropic properties. Based on previous studies [86, 87], we used a Young’s modulus of 200 GPa and a Poisson’s ratio of 0.278. Material properties of the brain were assumed to be nonlinear and were modeled using a 1st order Ogden hyperelastic model (equation (1) with the strain-energy equation,

\begin{equation*}\begin{array}{*{20}{c}} {\Psi = \frac{{{\mu _1}}}{{{\alpha _1}}}\left( {\lambda _1^{ - {\alpha _1}} + \lambda _2^{ - {\alpha _1}} + \lambda _3^{ - {\alpha _1}} - 3} \right) + \frac{1}{{{d_1}}}{{\left( {J - 1} \right)}^2}} \end{array}\end{equation*}

where ${\lambda _i}$ are the principal stretches, $J$ is the determinant of the elastic deformation gradient, and ${\mu _1}$, ${\alpha _1}$, and ${d_1}$ are the material properties. Budday *et al* previously characterized material properties of human cortical tissue using this model [94]. Namely, ${\mu _1} = 150.5{\text{ Pa}}$, ${\alpha _1} = 19$, and ${d_1} = 6.65 \times {10^{ - 5}}{\text{ }}{\text{P}}{\text{a}^{ - 1}}$.

#### 2.1.3. Boundary conditions

We defined boundary conditions to model a generalized relative micromotion between the array and tissue. For each array geometry, the bottom face of the brain tissue, closest to the electrode tips, was spatially fixed (displacement of zero). The top face of the electrode array (opposite the brain tissue) was prescribed a displacement of 10 *µ*m in the *Y* direction (figure 1(b)). To verify our observations were not unique to our specific set of boundary conditions, additional models were run using differing displacement magnitudes (1 *µ*m, 5 *µ*m, 20 *µ*m, and 50 *µ*m) that correspond to the range of displacements that have been observed near the implant and encompass the range of displacements previously used in FEMs of single shank electrodes. Further models were run applying a 10 *µ*m displacement at varying angles relative to the *Y* axis (0°, 22.5° and 45°).

#### 2.1.4. Meshing

Meshes and finite element solutions were computed with ANSYS Mechanical 18.2 using a static structural problem type. While the micromotions we are modeling have a dynamic and oscillatory component, a static model provides computationally efficient first-order approximations of the strain surrounding the electrodes and can be used to observe spatial differences in the strain fields across the array. Both the array and brain were meshed using 2nd order tetrahedrons. Spheres of influence at the tip of each electrode were generated to refine the mesh near the shank tip. The sphere of influence was set to a radius of 10 *µ*m and constrained the mesh in this volume to have a maximal edge length of 1 *µ*m. This parameter choice was validated through a mesh convergence study. The full model for the 10 × 10 array and brain had a median element volume of 1.96 *µ*m^3^ (IQR = 0.26 *µ*m^3^–1014 *µ*m^3^).

#### 2.1.5. Simulations and strain analysis

For each array geometry and applied boundary condition, von Mises strain was computed at each node. Von Mises strain is a scalar value that provides a single measure that incorporates axial and shear strains. Exported nodal displacements, strains, and locations were analyzed using MATLAB 2022a. Our region of interest consisted of all nodes within 50 *µ*m of the electrode tips, representing the approximate volume of cortical tissue where single units can be recorded [95, 96]. Maximum strain was defined as the maximal nodal strain within this ROI. To obtain a more representative metric over the entire ROI, nodal strains were averaged over 2.5 *µ*m bins extending out from the electrode tip. Binned strains were then normalized by the volume they occupied to compute the average strain in the 50 *µ*m sphere. Electrodes were grouped based on the number of rows away from the edge of the array, generating a series of concentric electrode ‘rings’. Corner electrodes were separated into their own group. Electrodes were also analyzed based upon their distance from the center of the array.

#### 2.1.6. Mesh convergence

Mesh convergence was conducted to ensure our finite element solutions were mesh-independent. To improve computational efficiency, the mesh convergence study was carried out using a simplified array geometry with only one electrode shank. Meshes were refined using a 10 *µ*m sphere of influence surrounding the electrode tip with maximal element edge sizes of 3 *µ*m, 2 *µ*m, 1 *µ*m, 0.5 *µ*m, 0.3 *µ*m, and 0.2 *µ*m. Material properties and boundary conditions described previously were used. Average von Mises strain surrounding the electrode was then computed for each mesh. Maximum element edge size of 1 *µ*m was chosen as a tradeoff between accuracy and computational load, with less than 5% error compared to the finest mesh tested (supplementary figures 1(a) and (b)).

### 2.2. Neural data processing

#### 2.2.1. Human motor and somatosensory arrays

Data collected from two clinical study participants (P2 and P3) with implanted Utah arrays (*n* = 4 motor arrays, *n* = 4 somatosensory arrays; somatosensory arrays were used for microstimulation) was previously published by Flesher *et al* and others [9, 19, 25, 97]. For P2, 10 × 10 motor arrays were platinum and 10 × 6 somatosensory arrays were SIROF (sputtered iridium oxide film) coated. For P3, both 10 × 10 motor arrays and 10 × 6 somatosensory arrays were SIROF coated. Data were collected as part of an ongoing clinical trial of a sensorimotor BCI (NCT01894802) and conducted under an FDA Investigational Device Exemption with approval from the University of Pittsburgh Institutional Review Board. Methods for surgical implantation have been described previously [98–100]. Due to hardware limitations, recordings were not collected on all electrodes. For P2 10 × 10 motor arrays, three electrodes in each corner were unconnected. For P3 10 × 10 motor arrays, the four corner electrodes were unconnected. For all 10 × 6 somatosensory electrodes, connected electrodes form a checkerboard pattern. Impedance measurements at 1 kHz were taken using the NeuroPort signal processor (Blackrock Neurotech). SIROF-coated electrodes with impedances above 1 MΩ and platinum electrodes with impedances above 2 MΩ were deemed nonfunctional and excluded from subsequent analysis.

Spontaneous electrophysiological recordings were taken at 30 kHz during a restful period where the participants relaxed in a quiet room. Extraction of waveforms were similar to previous methods by Hughes *et al* [19]. Electrophysiological recordings were filtered using a 2nd order bandpass Butterworth filter with cutoff frequencies of 300 Hz and 5000 Hz. Recorded action potentials were detected using a threshold of 4.5 times the root-mean-square below the mean. Waveform snippets were generated by extracting a window 0.5 ms before and 1 ms after threshold crossing. The peak-to-peak voltage (PTPV) of each snippet was calculated by subtracting the minimum voltage from the maximum voltage. For each active electrode, an average was taken of the largest 2% of snippets to obtain a representative PTPV value. Only the largest snippets were used in order to estimate the maximum PTPV voltage that could be achieved. SNR was computed by dividing the PTPV for that electrode by the noise floor, which was computed as twice the standard deviation of the recording after removing the snippets. Five successive sessions were analyzed for each time period of interest (1 month, 1 year, and 2 years post-implantation). For each array, impedance, PTPV, and SNR measures were first averaged across the five sessions before normalizing within the individual array (supplementary figures 2 and 3). Electrodes deemed nonfunctional from our applied impedance criteria were removed prior to averaging and normalization. Session-to-session variability was assessed by analyzing the electrode standard deviation across the five successive sessions. We observed that edge electrodes had reduced session-to-session variability compared to interior electrodes for impedance and PTPV measures at 1 year post-implantation (supplementary figure 4).

We also assessed the similarity in bandpass-filtered recordings between neighboring electrodes. For each electrode, its bandpass-filtered recording was correlated with the bandpass-filtered recording on all neighboring electrodes (3–8 electrodes depending on location). The average of these 3–8 neighboring electrode correlations was then computed. These values were averaged within an array across five successive recording sessions for each time period. The average correlation values could theoretically vary between −1 and 1. However, due to the large number of positive correlations present, all averaged correlations were greater than zero.

#### 2.2.2. NHP area V4 arrays

A second dataset from Utah arrays implanted in NHPs was also analyzed (*N* = 2 animals, *n* = 4 area V4 arrays) [27, 101]. For both animals, 8 × 8 V4 arrays were iridium-oxide coated. Methods for surgical implantation and data collection have been described previously. Impedance measurements at 1 kHz were taken using the Impedance Tester in the Research Central Software Suite (Blackrock Neurotech). Visually evoked electrophysiological recordings were taken during a visual stimulation detection task. Snippet extraction and PTPV calculations were conducted in the same manner as with the human array data [19, 27]. Envelope multi-unit activity (eMUA) was also extracted as previously described [102]. Evoked SNR was computed by subtracting the average eMUA from baseline spontaneous activity from the peak trial-averaged eMUA during stimulation, and then dividing by the standard deviation of the baseline eMUA. Impedance, PTPV, and SNR measures were normalized within each individual array (supplementary figure 5). Electrodes deemed nonfunctional from our applied impedance criteria were removed prior to averaging and normalization. Due to limited number of recording sessions, session-to-session variability was not assessed.

### 2.3. Statistics

Statistical analyses were performed in MATLAB 2022a. Means across groups from the modeled data were compared using one-way ANOVA with Tukey’s HSD post-hoc testing. For electrophysiological data, linear regression and ANOVA assumptions of normality and homoscedasticity were not met across electrode location groups. Therefore, Kruskal–Wallis tests with post-hoc Dunn’s tests were used to determine whether a significant difference in medians across multiple groups exists. Similarly, linear correlation assumptions were violated when assessing the relationship between electrophysiological measures and modeled strain. As a result, Spearman’s rank-order correlation was conducted to test for significant correlation. For significant correlations (*p* < 0.05) Spearman’s correlation coefficient (*ρ*) values are reported as a measure of the strength of the relationship. These values range from −1 to +1.

## 3. Results

We sought to determine the relationship between Utah array geometry, tissue strain, and the recording capabilities of these devices. We used FEMs to simulate micromotion-induced strain in brain tissue surrounding Utah arrays of various geometries, including 10 × 10, 10 × 6, and 8 × 8 grid configurations, to replicate those used in electrophysiological recordings. We then correlated these strain distributions with electrophysiological performance metrics—impedance, PTPV, and SNR—collected from Utah arrays implanted in both humans and NHPs.

### 3.1. FEM predicts micromotion-derived tissue strains at the edge of Utah arrays are greater than strains in the interior

We first asked how tissue strain varies from electrode to electrode across the Utah array. To this end, we used a FEM to predict the tissue strains resulting from micromotions in the brain (figures 1(a) and (b)). Contour plots of the resulting von Mises strain for the 10 × 10 array are shown in figure 1(c). We qualitatively observed increased tissue strains near the corner electrodes compared to the edge electrodes, both of which were greater than near interior electrodes (figure 1(d)). A mesh convergence study confirmed that the solution was independent of the mesh size (supplementary figures 1(a) and (b)).

**Figure 2.**
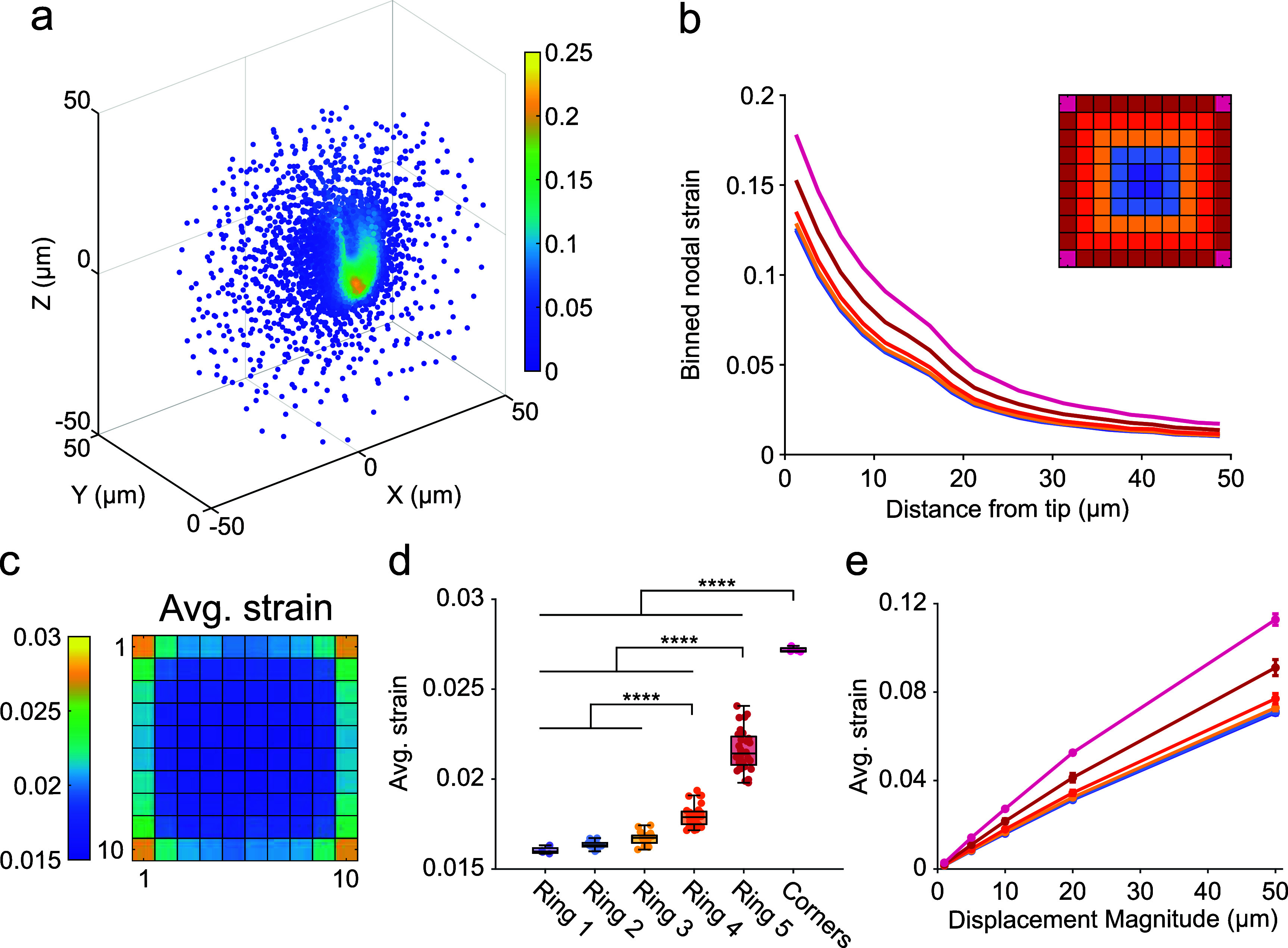
Modeled tissue strains arising from micromotions are greater on edge electrodes compared to interior electrodes. (a) Von Mises strains for brain tissue nodes within 50 *µ*m of the electrode tip. Example shown is a corner electrode. (b) Average spatial profile of strain surrounding electrodes in 10 × 10 array. Inset is color legend defining concentric electrode rings. The corner electrodes are designated as a distinct group. (c) Heatmap of average von Mises strain occurring within 50 *µ*m of each electrode. (d) Average strains at corner, ring 5, and ring 4 electrodes were significantly different from all other groups (*p* < 0.0001, one-way ANOVA with Tukey’s post-hoc test). (e) Average strains within electrode groups plotted as a function of applied displacement magnitude. Error bars are standard deviations. **** *p* < 0.0001.

A sphere with radius 50 *µ*m centered at each electrode tip was defined as our region of interest (figure 2(a)). To obtain a strain metric that accounts for the entire ROI without bias from mesh density variations, nodal strains were averaged over 2.5 *µ*m bins extending out from the electrode tip (figure 2(b)). These binned strains were then normalized by the volume they occupied to calculate the average strain within the 50 *µ*m sphere. Distinct separation between the strain curves for corner, edge, and interior electrodes was observed. When assessing the average strain for individual electrodes, tissue near corner electrodes showed significantly higher strains compared to other edge electrodes, and edge electrodes had greater strains than interior electrodes (*p* < 0.0001, one-way ANOVA with Tukey’s post-hoc, figures 2(c) and (d)). Additionally, the 2nd and 3rd outermost rings in the 10 × 10 array exhibited greater strains than the other interior electrodes. Similar trends were observed when analyzing the maximum strain within the ROIs, with tissue surrounding corner and edge electrodes showing significantly higher maximal strains compared to interior regions (*p* < 0.0001, one-way ANOVA with Tukey’s post-hoc, supplementary figure 6). Consistent results in the average strains surrounding 10 × 6 and 8 × 8 Utah arrays indicate that the spatial patterns were a consequence of the planar geometry of the array and not the number of electrodes. Further analysis revealed that grouping electrodes by concentric rings better distinguished strain differences than using distance from the array center (supplementary figures 7(a) and (b)).

We next asked whether the magnitude or direction of the displacement impacted the observed spatial trends. Using the 10 × 10 arrays, additional models were run using displacements of 1 *µ*m, 5 *µ*m, 20 *µ*m, and 50 *µ*m along the *Y* direction (supplementary figure 8(a)). As expected, the predicted strain increased with the displacement magnitude. For all displacements tested, we observed increased strain surrounding the edge electrodes compared to the interior electrodes (figure 2(e)). We next ran models applying a 10 *µ*m displacement at 0°, 22.5° and 45° relative to the *Y* axis (staying in the *XY* plane). Some qualitative differences were observed whereby the edge and corner electrodes that aligned to the angle of displacement experienced greater strains than the off-axis corner electrodes. However, regardless of the angle of displacement, strain on the edge and corner electrodes was on average greater than on interior ones (supplementary figures 8(b) and (c)). Overall, we find that the elevated strains on edge electrodes were preserved across different displacement angles and physiologically relevant magnitudes. These findings highlight the importance of electrode placement and array design in influencing tissue strain profiles, which can potentially impact overall device performance and tissue health.

### 3.2. Modeled tissue strains are negatively correlated with impedance across the lifetime of the implant

Given that electrode placement and array design influence tissue strain, we next examined whether our modeled strain magnitudes were correlated with 1 kHz impedance measurements from previously implanted array studies [9, 19, 27, 97, 101]. Recordings were obtained from Utah arrays implanted in human study participants (*N* = 2 participants, *n* = 8 arrays) at approximately 1 month, 1 year, and 2 years post-implantation. The 10 × 10 arrays (*n* = 4) were implanted in the motor cortex while the 10 × 6 stimulating arrays (*n* = 4) were implanted in Area 1 of the somatosensory cortex. Similarly, recordings from 8 × 8 arrays implanted in the V4 region of macaques (*N* = 2 animals, *n* = 4 arrays) were collected at approximately the same time points.

**Figure 3.**
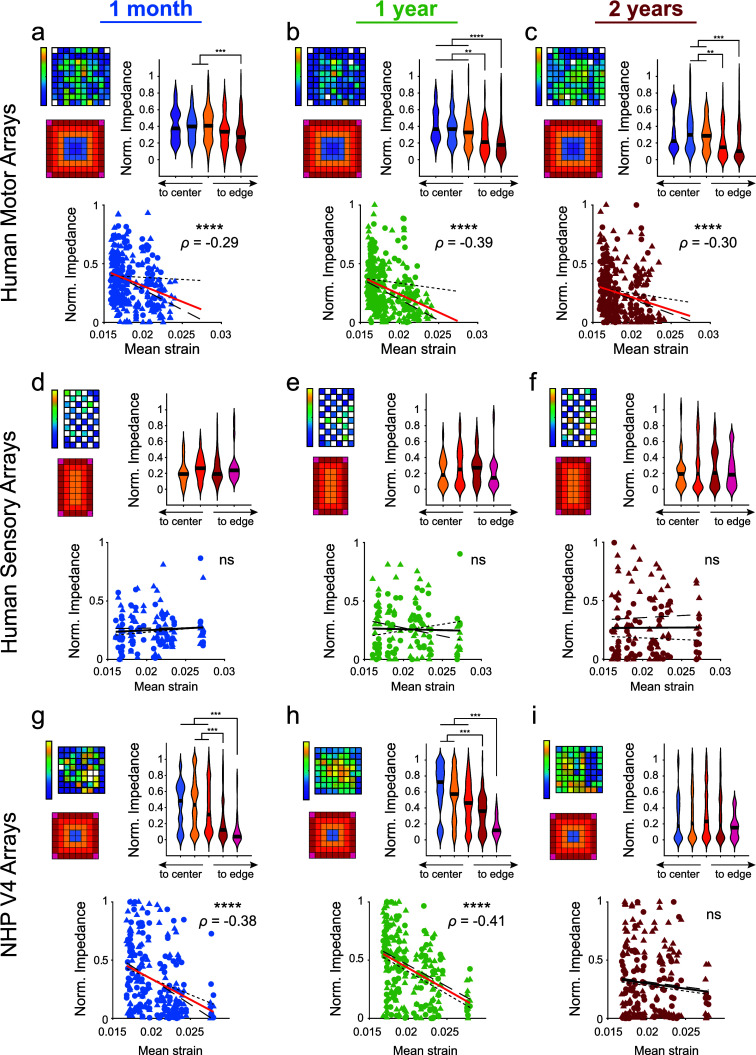
Impedance of implanted Utah arrays are negatively correlated with predicted micromotion strains. (a)–(c) Normalized 1 kHz impedance measured approximately 1 month, 1 year, and 2 years post-implantation in motor cortex of human study participants (*N* = 2 participants, *n* = 4 arrays). Heatmap shows an example array. Violin plots show edge electrodes had reduced impedances compared to more interior shanks at each time point (Kruskal–Wallis test with post-hoc Dunn’s test). Normalized impedances were correlated with modeled von Mises strains using Spearman’s rank-order correlation. Spearman’s correlation coefficient (*ρ*) values reported for significant correlations (*p* < 0.05). (d)–(f) Normalized 1 kHz impedance measured approximately 1 month, 1 year, and 2 years post-implantation in somatosensory cortex of human study participants (*N* = 2 participants, *n* = 4 arrays). Heatmap shows an example array. Violin plots showed impedance did not vary between edge and interior electrodes. (Kruskal–Wallis test with post-hoc Dunn’s test). Normalized impedances were correlated with modeled von Mises strains using Spearman’s rank-order correlation. No significant correlations observed. (g)–(i) Normalized 1 kHz impedance measured approximately 1 month, 1 year, and 2 years post-implantation in area V4 of macaque monkeys (*N* = 2 animals, *n* = 4 arrays). Heatmap shows an example array. Violin plots show edge electrodes had reduced impedances compared to more interior shanks at 1 month and 1 year post-implantation (Kruskal–Wallis test with post-hoc Dunn’s test). Normalized impedances were correlated with modeled von Mises strains using Spearman’s rank-order correlation. Spearman’s correlation coefficient (*ρ*) values reported for significant correlations (*p* < 0.05). ** *p* < 0.01, *** *p* < 0.001, **** *p* < 0.0001. Black line in violin plots show median value. P2 and Monkey L data is plotted with circles and small-dashed trend line. P3 and Monkey A data is plotted with triangles and large-dashed trend line. The thick trend line fits the combined data.

Impedance values were normalized within individual arrays by subtracting the minimum impedance from all electrodes and then dividing by the maximum. For 10 × 10 human motor arrays and 8 × 8 NHP V4 arrays, electrodes near the edge of the array had reduced impedance compared to those in the interior (Kruskal–Wallis test with post-hoc Dunn’s test). Modeled strains were then correlated with the normalized impedances using Spearman’s rank-order correlation. For the 10 × 10 arrays, a significant negative correlation (*p* < 0.0001) was found for each time period (figures 3(a)–(c)), with the highest magnitude Spearman’s *ρ* of −0.39 occurring at 1 year post-implantation (figure 3(b)); higher strain was associated with lower impedance. No significant correlation was observed for the 10 × 6 arrays at any time point (figures 3(d)–(f)). Finally, for the 8 × 8 arrays, significant negative correlations (*p* < 0.0001) were found at 1 month and 1 year after implantation, both having comparable Spearman’s *ρ*, −0.38 and −0.41, respectively (figures 3(g)–(i)). These results suggest that impedance can vary with strain, but the relationship is dependent on array geometry and implantation site.

**Figure 4.**
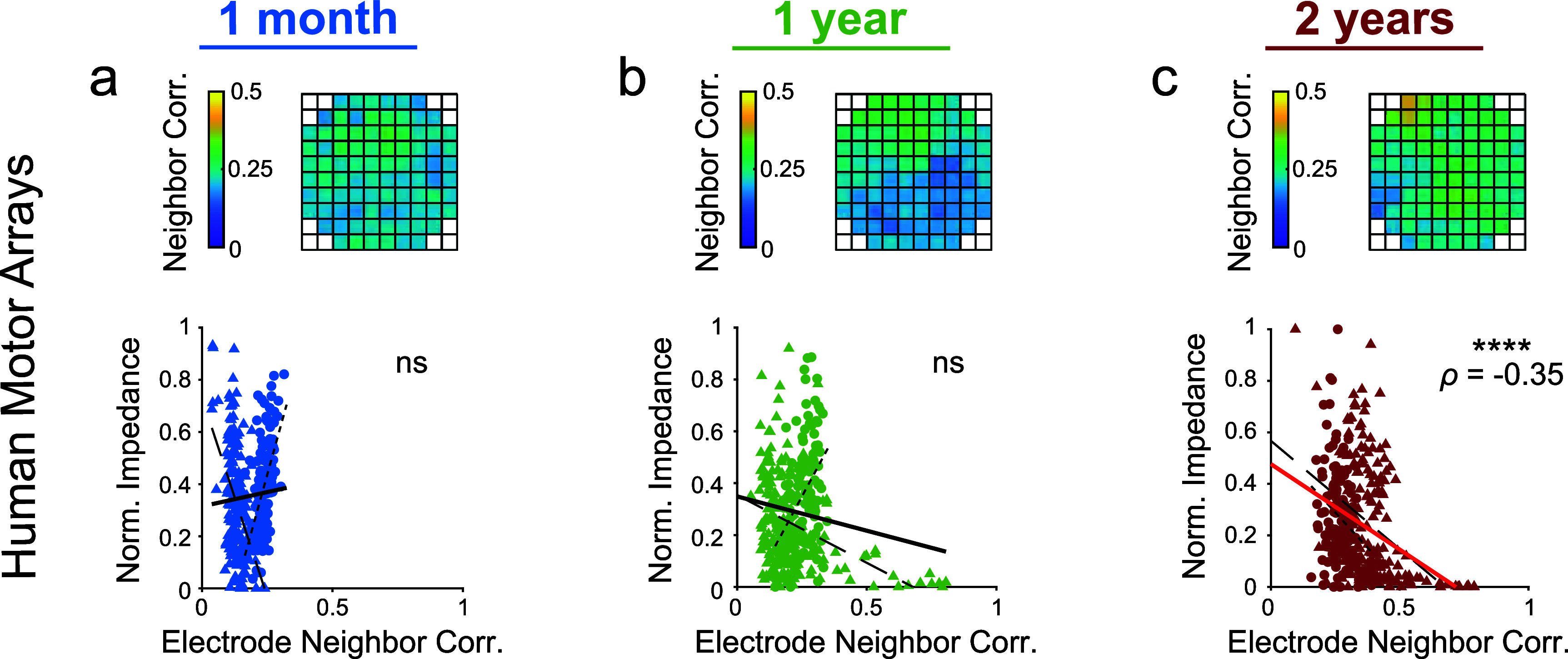
Similarity in bandpass-filtered electrophysiological recordings between neighboring electrodes correlates with impedance at 2 years post-implantation in human motor arrays. Using arrays implanted in human motor cortex (*N* = 2 participants, *n* = 4 arrays) at (a) 1 month, (b) 1 year, and (c) 2 years post-implantation, bandpass-filtered electrophysiological recordings from each electrode were correlated to the bandpass-filtered electrophysiological recordings from all surrounding electrodes. An average correlation was computed for each electrode, estimating its similarity to surrounding electrodes. Heatmap shows an example array. Normalized 1 kHz impedance was then correlated with the average neighborhood correlation for that electrode using Spearman’s rank-order correlation. Spearman’s correlation coefficient (*ρ*) values reported for significant correlations (*p* < 0.05). **** *p* < 0.0001. P2 data is plotted with circles and small-dashed trend line. P3 data is plotted with triangles and large-dashed trend line. The thick trend line fits the combined data.

An alternate hypothesis as to why edge electrodes have reduced impedance compared to interior electrodes could be failure of insulation on the back of the array occurring more frequently on the edge of the array compared to the interior. This would lead to lowered electrical resistance between neighboring electrodes, which could contribute to reduced impedances on individual electrodes. Lower resistance between neighboring electrodes should cause higher correlations in their electrophysiological recordings. To test this alternate hypothesis for the 10 × 10 human motor arrays, correlations in the bandpass-filtered electrophysiological recordings between neighboring electrodes were computed and then correlated to the collected impedance data (figures 4(a)–(c)). We found a significant correlation only at 2 years post-implantation (figure 4(c)). Therefore, this insulation failure does not explain the reduced edge electrode impedance relative to interior electrodes observed at 1 month and 1 year post-implantation. Interestingly, for one 10 × 10 human motor array, very high electrode neighbor correlations were observed at one edge of the array and highly correlated to low impedance values (supplementary figures 9(b) and (c)). This suggests that insulation failure at the edge of the array does impact the electrode impedance, but other factors, such as strain, may have larger effects, especially in the first year following implantation.

### 3.3. Modeled tissue strains are negatively correlated with spontaneous PTPV and SNR in human motor arrays years after implantation

**Figure 5.**
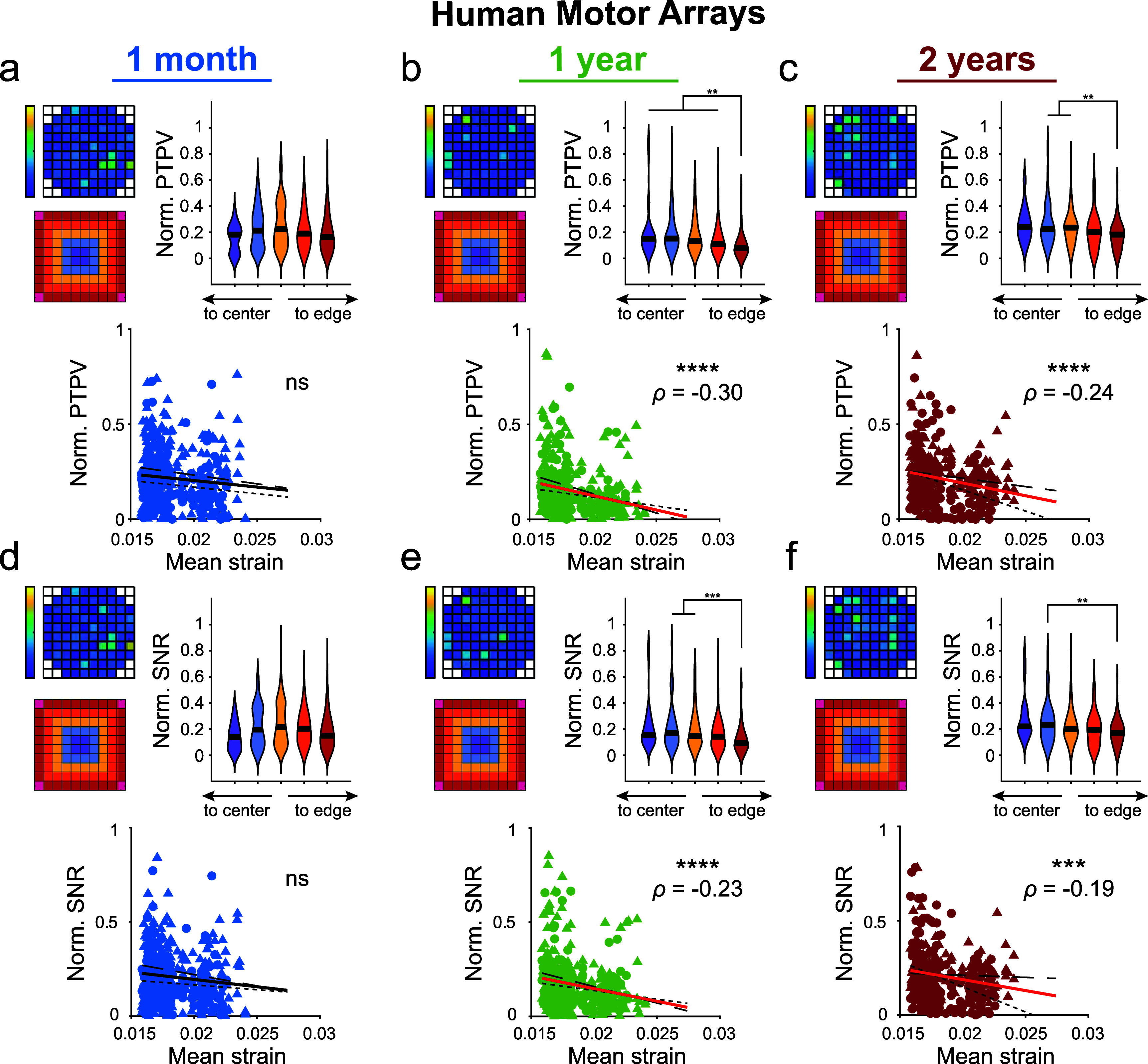
Spontaneous peak-to-peak voltage and SNR from implanted human motor arrays are negatively correlated with predicted micromotion strains. (a)–(c) Normalized PTPV measured at 1 month, 1 year, and 2 years post-implantation in motor cortex of human study participants (*N* = 2 participants, *n* = 4 arrays). Heatmap shows an example array. Violin plots show edge electrodes had reduced PTPVs compared to more interior shanks at 1 year and 2 years post-implantation (Kruskal–Wallis test with post-hoc Dunn’s test). Normalized PTPVs were correlated with modeled von Mises strains using Spearman’s rank-order correlation. Spearman’s correlation coefficient (*ρ*) values reported for significant correlations (*p* < 0.05). (d)–(f) Same as above, but using normalized SNR measured at 1 month, 1 year, and 2 years post-implantation in motor cortex of human study participants. Violin plots show edge electrodes had reduced SNRs compared to more interior shanks at 1 year and 2 years post-implantation (Kruskal–Wallis test with post-hoc Dunn’s test). Normalized PTPVs were correlated with modeled von Mises strains using Spearman’s rank-order correlation. Spearman’s correlation coefficient (*ρ*) values reported for significant correlations (*p* < 0.05). ** *p* < 0.01, *** *p* < 0.001, **** *p* < 0.0001. Black line in violin plots show median value. P2 data is plotted with circles and small-dashed trend line. P3 data is plotted with triangles and large-dashed trend line. The thick trend line fits the combined data.

Next, we investigated whether our modeled strains were correlated with the quality of recorded spontaneous neural activity from human motor and somatosensory arrays. In the motor cortex, edge electrodes had lower PTPV and SNR compared to interior electrodes at 1 year and 2 years post-implantation (Kruskal–Wallis test with post-hoc Dunn’s test). We also observed a significant negative correlation between strains and both spontaneous PTPV and SNR at 1 year and 2 years post-implantation (*p* < 0.001, figures 5(b), (c), (e) and (f)). While insulation failure could explain this trend at 2 years post-implantation, the relationship in the first year could be the result of elevated strains reducing the ability of the electrodes to record nearby neurons. In contrast, no significant correlation between strain and PTPV or SNR was found in the somatosensory arrays. (*p* > 0.05, supplementary figures 10(a)–(f)). The limited number of active channels recorded in these arrays may account for the weaker relationships observed. This highlights the complex interplay between mechanical strain, electrode performance, and the longevity of neural recordings.

### 3.4. Modeled tissue strains are positively correlated with envelope MUA-derived evoked SNR in NHP V4 implants

**Figure 6.**
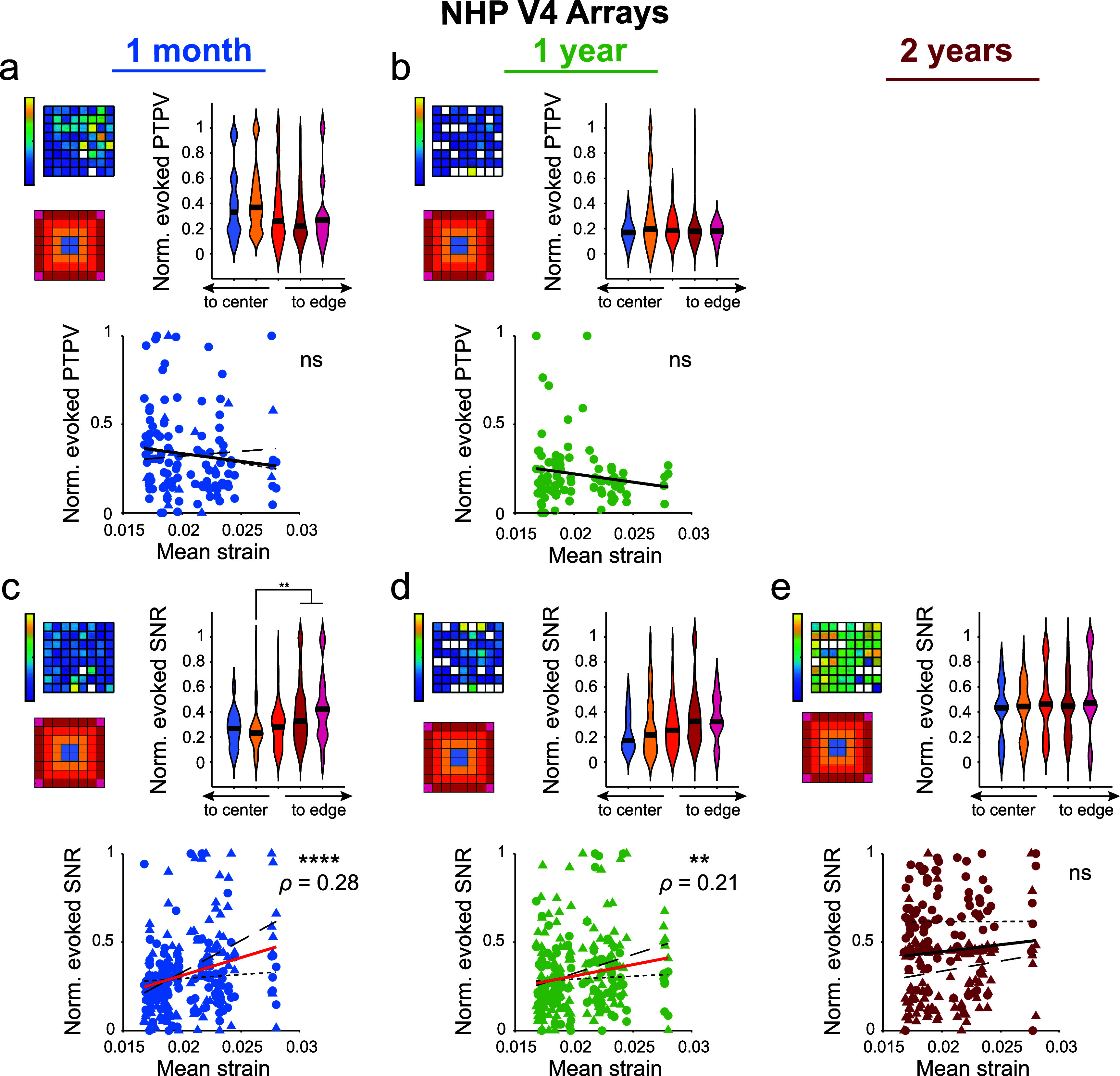
Visually evoked SNR from eMUA recorded from NHP Utah arrays are positively correlated with predicted micromotion strains. (a) and (b) Normalized PTPV measured at 1 month and 1 year post-implantation in area V4 of macaque monkeys (*N* = 2 animals, *n* = 4 arrays). Heatmap shows an example array. Violin plots show PTPV did not vary between edge and interior shanks (Kruskal–Wallis test with post-hoc Dunn’s test). Normalized PTPVs were correlated with modeled von Mises strains using Spearman’s rank-order correlation. No significant correlations observed. (c)–(e) Normalized SNR from eMUA measured at 1 month, 1 year, and 2 years post-implantation in NHP V4. Heatmap shows an example array. Violin plots show normalized eMUA SNR was greater on edge electrodes compared to interior shanks at 1 month post-implantation (Kruskal–Wallis test with post-hoc Dunn’s test). Normalized SNRs were correlated with modeled von Mises strains using Spearman’s rank-order correlation, with significant correlations occurring at 1 month and 1 year post-implantation. ** *p* < 0.01, *** *p* < 0.001, **** *p* < 0.0001. Black line in violin plots show median value. Monkey L data is plotted with circles and small-dashed trend line. Monkey A data is plotted with triangles and large-dashed trend line. The thick trend line fits the combined data.

Given that mechanical strain influences 1 kHz impedance and spontaneous neural recordings, we next investigated whether predicted tissue strains were correlated with our ability to record visually evoked neuronal activity. Analysis of the PTPV from threshold-crossing snippets showed no correlation with tissue strains in the NHP recordings (figures 6(a) and (b)). The PTPV data at 2 years post-implantation was excluded due to an excessive number of electrodes failing to register any threshold-crossing events. Consequently, we analyzed the envelope MUA and computed the evoked SNR during a stimulus detection task. This analysis revealed a significant positive correlation (*p* < 0.0001 at 1 month, *p* < 0.01 at 1 year) between the evoked MUA SNR and tissue strains at 1 month and 1 year post-implantation (figures 6(c)–(e)). Unlike the PTPV results, the positive correlation between MUA SNR and tissue strains suggests that higher mechanical strains are associated with improved evoked SNR, indicating a complex relationship between strain and the quality of visually evoked neural recordings.

## 4. Discussion

This study aimed to model the strain profiles in tissue surrounding chronically implanted electrodes caused by micromotions and to determine whether these strains correlate with functional metrics from implanted arrays. Utah arrays have been a standard recording modality for human and NHP BCIs for decades. However, the impact of array geometry on neuronal recording remains poorly understood. Here, we establish a spatial dependence for tissue strain and array recording capabilities, specifically noting differences between edge and interior shanks. Insights into this relationship are essential for developing improved multi-electrode arrays that minimize strains surrounding electrodes or placement strategies for serially implanted multi-shank electrodes to optimize the shielding effects of the edge electrodes. These innovations will help support the goal of enhancing device longevity and advancing the clinical translation of motor and sensory BCIs.

### 4.1. Signal quality in human motor arrays is negatively correlated with predicted tissue strains

We observed a significant (*p* < 0.001) negative correlation between our modeled tissue strain and the PTPV and SNR from spontaneous recordings in human motor cortex (figures 5(b), (c), (e) and (f)). The presence of correlation after 1 year and lack of correlation at 1 month may be attributed to increased glial scarring surrounding edge electrodes, a consequence of elevated micromotion-derived strains. Such scarring fosters a neurodegenerative environment near the electrode, diminishing the efficacy of recording and stimulating devices [103–105]. Both microglia and astrocytes can respond to mechanical strains and stiffness gradients in the tissue [43, 53–55, 58, 59, 72–76, 106, 107]. Microglia have been shown to migrate towards stiffer substrates and are more prone to display activated macrophage-like phenotypes in these stiffer environments [55, 106, 108]. For example, in Alzheimer’s disease models, microglia migration to amyloid beta plaques is mediated by mechanosensitive ion channels [58, 59]. Mechanical stimuli can similarly contribute to astrocyte reactivity via the opening of mechanosensitive ion channels [55, 73, 107]. The heightened strain experienced by corner and edge electrodes (figure 2(c)) likely contributes to increased glial reactivity. The heightened strain near edge electrodes may also contribute to preferential damage to edge electrodes directly, impairing their ability to detect neuronal activity. Studies analyzing explanted Utah arrays support these findings. Notably, Patel *et al* reported more frequent tip breakage and coating cracks on edge electrodes compared to interior shanks, aligning with our model’s prediction of greater strain at edge electrodes of an array [109–111].

At early time points, strains exerted on blood vessels near the edge of the Utah array can also contribute to vascular damage and subsequent ischemia in the central regions of the array. High mechanical strain, especially near edge electrodes, can cause significant stress on adjacent blood vessels, leading to endothelial cell damage, vessel rupture, or increased permeability [80, 112]. Such damage can compromise the BBB and result in localized hemorrhage or edema. This vascular injury impairs oxygen and nutrient delivery to the brain tissue, potentially leading to ischemic conditions in downstream regions. The observation of increased strain near edge electrodes (figure 2(c)) could correlate with heightened vascular stress and damage in those areas. Consequently, the compromised blood supply near the edge can affect the central regions of the array, resulting in ischemia. This ischemic environment can severely impact neuronal health and functionality, thereby affecting the quality of neural recordings. For instance, the lack of correlations observed at 1 month post-implantation (figures 5(a) and (d)) might be attributed to ischemic damage and silencing neuronal activity in these central regions [52]. However, over the long term, vessels may repair and revascularize the tissue, evidenced by increased PTPV and SNR for central electrodes compared to edge electrodes at 1 year and 2 year post-implantation. These findings underscore the impact of mechanical strain on neural recording quality, particularly highlighting how increased strain around edge electrodes correlates with diminished recording performance.

### 4.2. SNR of evoked eMUA in NHPs is positively correlated with predicted strains

In our analysis of visually evoked recordings from NHP V4, we observed an unexpected positive correlation between predicted strain and the evoked SNR of envelope MUA (figures 6(c) and (d)). This finding suggests that higher predicted strains are associated with better quality evoked neural signals. The envelope MUA, which aggregates activity from multiple neurons, captures signals from a broader neuronal population than single-unit recordings, potentially from regions beyond the immediate vicinity of the electrode [113–115]. Our model indicates minimal strain variation beyond 40 *µ*m from the electrode tip (figure 2(b)), suggesting that micromotion strains predominantly affect neurons very close to the electrode. Consequently, the observed positive correlation between strain and MUA might reflect that the MUA detects signals from relatively unaffected, healthier tissue further from the electrode. This implies that other factors related to the array geometry or the nature of the evoked response might be influencing the positive correlation.

For instance, the array’s design could enhance signal detection from neurons located in less strained regions or affect the spatial distribution of strain in a way that benefits broader neuronal populations. Additionally, elevated strains on edge electrodes could improve adhesion of tissue compared to interior electrodes in the early months after implantation, contributing to improved device integration and the observed positive correlation between strain and SNR at 1 month post-implantation. Alternatively, the positive correlation could be the result of differences in the extra-axial intracranial space between humans (3–7 mm) and macaques (0–2 mm) [116]. The reduced space in macaques may lead to increased mechanical compression of the array compared to humans, increasing the scarring and encapsulation of the electrodes. However, taken together, the positive correlation between predicted strain and MUA in NHP V4 highlights a complex interaction between mechanical strain and the quality of evoked neural recordings, suggesting that higher strain may enhance signal capture from a broader neuronal population, possibly due to improved electrode–tissue integration or other geometric factors related to the array design.

Also note that a previous study assessing the chronic performance of these NHP arrays showed that by 3 years after implantation, on several arrays, greater tissue encapsulation was observed on electrodes located at the center of the arrays (e.g. Figure 6(b) of Chen *et al* [27]), which may contribute to lower SNR in centrally located electrodes. While we predict that elevated strains on edge electrodes would increase scarring along the edge, deviations from this expectation within these V4 arrays may be explained by the specific implantation methods used by Chen *et al* [27] that differed from the standard approaches used in previous studies (both clinical and pre-clinical). Specifically, the high density of implanted arrays may have created interaction effects altering the tissues strains, and the use of tissue glue to secure the arrays could have altered typical micromotions as the glue bound neighboring arrays and wire bundles in a large platform. This underscores that Utah array implantation methods may greatly modulate the chronic tissue response to micromotion-induced strains and impact how elevated strain can contribute to device function.

### 4.3. Impedance is negatively correlated with predicted tissue strain across the lifetime of the array

Analyzing impedance measurements provides insights into the electrode–tissue interface, revealing how mechanical strains influence electrode performance. We observed a significant negative correlation between impedance and predicted micromotion-derived strains in both human motor and NHP V4 arrays (figures 3(a)–(c) and (g)–(i)). This correlation indicates that as predicted strain increases, impedance decreases, highlighting the impact of mechanical strain on electrode functionality. Interestingly, we did not observe any significant trend when assessing the human S1 arrays (figures 3(d)–(f)). One explanation is that the rectangular 10 × 6 geometry limits the number of interior electrodes compared to the 10 × 10 and 8 × 8 arrays, which could impact the amount of strain shielding that occurs. Additionally, the reduced number of connected electrodes also limited the amount of data that could be analyzed compared to the human motor and NHP V4 arrays.

Over time, we noted a consistent decrease in impedance, aligning with findings from previous studies [27, 59, 87, 117–119]. This trend may be attributed to increasing fluid accumulation in the peri-electrode space. Elevated fluid levels could result from the mechanical stresses and strains experienced by the electrodes, particularly those located at the edges. This fluid buildup might be due to the gliotic response to the fluid shear stress near the electrode, which is mediated by mechanosensitive ion channels [43]. Mechanosensitive ion channels, such as Piezo1, play a role in detecting mechanical forces and responding to changes in the local environment, including fluid shear stress [43, 54]. The increased micromotion strain around edge electrodes might exacerbate fluid accumulation and enhance the gliotic response, leading to a reduction in impedance. This reduction reflects a deterioration in the electrode–tissue interface, where increased fluid accumulation can disrupt the effective electrical coupling between the electrode and surrounding neural tissue. Additionally, the fluid filled space could alter the transport and diffusion of extracellular species necessary for metabolism [120–124] which could also contribute to varied neuronal health in surrounding tissue and recording capabilities across electrodes.

Increased mechanical strain could also contribute to the mechanical failure of the insulation material surrounding the electrode. The parylene insulation is designed to prevent electrical leakage and ensure stable impedance readings [34, 87, 125]. However, prolonged exposure to mechanical stress and strain can cause micro-cracks or delamination in the insulation layer [125]. These defects can compromise the insulating properties, leading to increased electrical leakage and a subsequent decrease in impedance. As the insulation material degrades, its ability to maintain a stable interface between the electrode and the tissue diminishes, further exacerbating impedance reduction [34]. Taken together, the negative correlation between impedance and predicted strain underscores the relationship between mechanical strain and electrode performance over time. As strain increases, impedance decreases, adversely impacting electrode performance and electrode–tissue interface.

### 4.4. Predicted spatial differences in tissue strain arise from planar geometry and material properties of Utah arrays

Previous work has modeled tissue strain surrounding linear electrode implants [85–91], often focusing on how soft and flexible electrodes could reduce strain compared to traditional silicon probes. These models indicated that soft electrodes might lower strain and scarring [86]. Research on linear probes has also highlighted the importance of electrode site geometry on recording performance [87]. While such probes are common in rodent studies, human and NHP BCI research predominantly uses planar Utah arrays for cortical access. While FEM has explored temperature dissipation in these arrays [126, 127], the impact of neighboring shanks in Utah arrays on tissue strain due to micromotion has not been previously assessed.

Similar to previous studies [85, 86], our model used a linear displacement of the arrays with fixed brain tissue to simulate a generalized relative motion (figure 1(b)). In an effort to be more consistent with current studies using *ex vivo* mechanical testing of cortical tissue, we applied a nonlinear constitutive model for the brain tissue [94]. Contour plots showed increased von Mises strain at the edges and corners of the array compared to interior electrodes (figure 1(d)). The symmetric nature of the contours arises from the bonded contact defined between the array and brain, simulating tissue adhesion on the electrode. This allows the electrode to apply both compressive and tensile strains on the tissue. Under a 10 *µ*m lateral displacement, we observe the strain profiles being to taper off near 20 *µ*m, with minimal change in strain observed beyond 40 *µ*m from the electrode. Previous work assessing microglial encapsulation of Utah arrays implanted in NHPs found the average microglial encapsulation thickness to be 16.1 *µ*m ± 10.0 *µ*m, corresponding to our observed strain profiles [128].

Further analysis indicated that corner electrodes caused greater strain than edge electrodes, which in turn imparted more strain than interior electrodes (figure 2(c)). Across the three array geometries, we found that the average modeled strains were similar (supplementary figure 7). The strain distribution was influenced by the array’s ring structure, with tissue near the edges experiencing higher strain compared to the center (figure 2(d)). Additionally, the observed spatial trends in predicted strains were conserved across varying displacement magnitudes and angles in the *XY* plane (figure 2(e), supplementary figure 8). This suggests that the Utah array’s planar geometry results in a shielding effect, where the interior tissue experiences less strain.

Modifying the Utah array’s geometry could enhance device performance. For instance, adding a new ring of nonfunctional shanks might improve strain shielding but could also increase cortical damage and insertion difficulty. Additionally, using softer or more flexible materials, as seen with linear arrays, could reduce mechanical mismatch and glial scarring [62–69, 129, 130]. Developing free-floating wireless arrays might also minimize tangential tethering forces, potentially reducing tissue strains [131]. New coatings could improve integration with the extracellular matrix and alter how forces affect nearby cells [132]. Taken together, future designs of planar arrays should therefore consider how changes in geometry and material properties impact the strain experienced by surrounding tissues.

### 4.5. Limitations

Many factors could influence the observed correlations, and this study does not establish tissue strain as a causal factor for array performance. Instead, it highlights how Utah array performance and the surrounding cellular environment vary with array geometry. Other contributing factors may include the metabolic support of neurons, which is crucial for survival near the electrode–tissue interface. Neurons on edge and corner electrodes might benefit from proximity to less damaged tissue, enhancing their survival compared to those in the array’s center.

The curvature of the brain could also affect how array geometry impacts recording performance. For electrocorticography grids, brain curvature influences the forces applied to cortical tissue [133]. A similar effect may occur with Utah arrays, where shank orientation relative to the brain surface could impact strain distribution. Additionally, the laminar structure of the cortex might lead to differences in neuronal layers recorded by edge versus interior electrodes, potentially contributing to the observed ring-structured performance correlations (figures 5(b), (c), (e) and (f)) [134, 135].

Insertion methods also play a role as demonstrated by linear arrays studies [29, 48, 77, 78, 81, 136]. Unlike linear arrays, Utah arrays are typically implanted using a pneumatic inserter, which introduces strain differently [137, 138]. A recent study showed insertion velocity affects strain near the tip but not along the shank [139]. Although this study did not assess array geometry, similar strain distributions might occur during insertion as observed with micromotions (figure 2(c)), potentially impacting performance.

Differences in recording conditions and surgical procedures between the human and NHP experiments limit our ability to draw clear comparisons between the arrays as many confounds may impact the observed correlations. These comparisons are further restricted by our limited sample size. Finally, this modeling study made critical assumptions about the array’s material properties and applied forces. We used a simplified displacement profile in this study to investigate a generalized micromotion between the array and tissue. However, future analyses should develop more specific models for key forces of interest, including tethering and vessel pulsations. Our model also did not account for insertion-induced strains, assuming initial tissue strain was zero. Our FEM also does not incorporate the temporal component of the displacement as we utilized a static model. Despite these limitations, the modeling provides a useful approximation, emphasizing that tissue conditions vary across array geometries and correlate with array performance.

## 5. Conclusion

Utah arrays are robust tools for recording neurons in intracortical BCI applications. Here, we establish how strain fields and recording performance can vary across multi-shank microelectrode arrays. Our FEM shows that tissue near edge and corner electrodes experience greater micromotion-derived strains compared to interior electrodes. These predicted strains are negatively correlated with impedances measured 1 month, 1 year, and 2 years after implantation. Additionally, strains are negatively correlated with SNR and PTPV of spontaneous neural activity recorded in human motor cortex while positively correlated with evoked SNR using eMUA from recordings in NHP area V4. Although tissue strain appears to be a significant factor affecting recording quality, other factors such as metabolic support may also play a role. Overall, our findings underscore that the geometry of the Utah array influences both electrode impedance and neural recording capabilities.

## Data Availability

The data that support the findings of this study are available upon reasonable request from the authors. Supplementary figures available at https://doi.org/10.1088/1741-2552/ae1bda/data1.
